# New approach methodologies to facilitate and improve the hazard assessment of non-genotoxic carcinogens—a PARC project

**DOI:** 10.3389/ftox.2023.1220998

**Published:** 2023-07-10

**Authors:** Marc Audebert, Ann-Sophie Assmann, Amaya Azqueta, Pavel Babica, Emilio Benfenati, Sylvie Bortoli, Peter Bouwman, Albert Braeuning, Tanja Burgdorf, Xavier Coumoul, Kloé Debizet, Maria Dusinska, Norman Ertych, Jörg Fahrer, Verena Fetz, Ludovic Le Hégarat, Adela López de Cerain, Harm J. Heusinkveld, Kevin Hogeveen, Miriam N. Jacobs, Mirjam Luijten, Giuseppa Raitano, Cynthia Recoules, Elise Rundén-Pran, Mariam Saleh, Iva Sovadinová, Martina Stampar, Lea Thibol, Céline Tomkiewicz, Ariane Vettorazzi, Bob Van de Water, Naouale El Yamani, Bojana Zegura, Michael Oelgeschläger

**Affiliations:** ^1^ INRAE: Toxalim, INRAE, INP-ENVT, INP-EI-Purpan, Université de Toulouse 3 Paul Sabatier, Toulouse, France; ^2^ Department Experimental Toxicology and ZEBET, German Centre for the Protection of Laboratory Animals (Bf3R) and Department Food Safety, BfR: German Federal Institute for Risk Assessment, Berlin, Germany; ^3^ Department of Pharmacology and Toxicology, School of Pharmacy and Nutrition, UNAV: University of Navarra, Pamplona, Spain; ^4^ RECETOX: RECETOX, Faculty of Science, Masaryk University, Brno, Czechia; ^5^ IRFMN: Istituto di Ricerche Farmacologiche Mario Negri—IRCCS, Milan, Italy; ^6^ INSERM: INSERM UMR-S 1124 T3S—Université Paris Cité, Paris, France; ^7^ UL-LACDR: Leiden Academic Centre for Drug Research, Leiden University, Leiden, Netherlands; ^8^ Health Effects Laboratory, NILU: The Climate and Environmental Research Institute, Kjeller, Norway; ^9^ Department of Chemistry, RPTU: Division of Food Chemistry and Toxicology, Kaiserslautern, Germany; ^10^ ANSES: French Agency for Food, Environmental and Occupational Health and Safety, Fougères Laboratory, Toxicology of Contaminants Unit, Fougères, France; ^11^ RIVM: National Institute for Public Health and the Environment, Bilthoven, Netherlands; ^12^ Radiation, Chemical and Environmental Hazards, UKHSA: UK Health Security Agency, Chilton, Oxfordshire, United Kingdom; ^13^ Department of Genetic Toxicology and Cancer Biology, NIB: National Institute of Biology, Ljubljana, Slovenia

**Keywords:** non-genotoxic carcinogens, NGTxC, new approach methodologies, NAM, PARC

## Abstract

Carcinogenic chemicals, or their metabolites, can be classified as genotoxic or non-genotoxic carcinogens (NGTxCs). Genotoxic compounds induce DNA damage, which can be detected by an established *in vitro* and *in vivo* battery of genotoxicity assays. For NGTxCs, DNA is not the primary target, and the possible modes of action (MoA) of NGTxCs are much more diverse than those of genotoxic compounds, and there is no specific *in vitro* assay for detecting NGTxCs. Therefore, the evaluation of the carcinogenic potential is still dependent on long-term studies in rodents. This 2-year bioassay, mainly applied for testing agrochemicals and pharmaceuticals, is time-consuming, costly and requires very high numbers of animals. More importantly, its relevance for human risk assessment is questionable due to the limited predictivity for human cancer risk, especially with regard to NGTxCs. Thus, there is an urgent need for a transition to new approach methodologies (NAMs), integrating human-relevant *in vitro* assays and *in silico* tools that better exploit the current knowledge of the multiple processes involved in carcinogenesis into a modern safety assessment toolbox. Here, we describe an integrative project that aims to use a variety of novel approaches to detect the carcinogenic potential of NGTxCs based on different mechanisms and pathways involved in carcinogenesis. The aim of this project is to contribute suitable assays for the safety assessment toolbox for an efficient and improved, internationally recognized hazard assessment of NGTxCs, and ultimately to contribute to reliable mechanism-based next-generation risk assessment for chemical carcinogens.

## Introduction

Cancer is one of the leading causes of death in Europe, with nearly 3 million new cases and 1.3 million deaths per year (www.ecris.jrc.ec.europa.eu) (Dyba et al., 2021). The importance of cancer prevention is recognized in the EU’s Chemicals Strategy for Sustainability Towards a Toxic-Free Environment, as well as in Europe’s Beating Cancer Plan (EC, 2023). These ambitious programs are challenged by the fact that cancer is a highly diverse and complex disease that affects various organs and involves multiple steps and mechanisms of progression that vary with cancer type and tissue affected. This complexity is also reflected in the different functional capabilities that cancer cells must acquire during malignant transformation, as described by the concept of “hallmarks of cancer” (Hanahan, 2022). Moreover, the vast majority of cancer-related deaths occurs at the late stages of carcinogenesis (i.e., invasive growth and metastasis) (Lyden et al., 2022).

The multiple causes of cancer can be divided into endogenous risk factors, comprising genetic predisposition, aging, hormone levels, stem cell division and DNA replication infidelity, and exogenous risk factors like (natural) radiation, lifestyle, infections and exposure to environmental chemicals. Environmental chemicals causing cancer can be grouped into genotoxic and non-genotoxic substances (NGTxCs). In a recent report by the European Environment Agency, it is estimated that such exogenous risk factors are responsible for a not negligible part of cancer cases (EEA, 2022). Interestingly, the International Agency for Research on Cancer (IARC) estimates that 10%–20% of human carcinogens classified as Class 1 may act as NGTxCs (Hernandez et al., 2009). A comprehensive knowledge of the carcinogenic potential of substances is urgently needed to minimize exposure and thus the risk of cancer. Genotoxic chemicals interact directly with DNA or interfere with the cellular machinery that maintains genomic integrity, leading to mutations and/or chromosomal alterations. In contrast, NGTxCs promote tumor formation through a variety of mechanisms and pathways without evidence of genotoxicity (Jacobs et al., 2020). To classify carcinogenic chemicals, the concepts of key characteristics (Smith et al., 2016; Guyton et al., 2018; Smith et al., 2020; Madia et al., 2021) or the hallmarks of environmental insults (Peters et al., 2021) have been proposed to link carcinogenic modes of action and the hallmarks of cancer concept.

In the different regulations, carcinogenicity is evaluated in long-term (2-year) exposure studies with rodents, in combination with *in vitro* and *in vivo* genotoxicity testing (OECD, 2016a; OECD, 2016b; OECD, 2018a; OECD, 2018b; Madia et al., 2019; OECD, 2022a; OECD, 2022b). However, long-term exposure rodent bioassays are time-consuming, costly, ethically questionable, and considered of limited relevance for humans. Indeed, some mechanisms induced by NGTxCs are potentially confounding biological and physiological species differences (Heusinkveld et al., 2020a). Since less than 5% of chemicals registered worldwide have been tested in long-term carcinogenicity rodent studies (Doe et al., 2019), there is an urgent need for a transition to new approach methodologies (NAMs). This allows for the inclusion of human-relevant *in vitro* and *in silico* methods that enable using recent advances in our understanding of diseases and employ the latest non-animal methods available in a modern safety assessment toolbox.

NGTxCs may have multiple modes of action, including epigenetic alterations, endocrine modulation, immune suppression, or continuous stimulation of inflammatory responses, sustained proliferation and migration (Hernandez et al., 2009). However, various carcinogenic processes are not exclusively cancer related, since they are also involved in non-cancer processes. In addition, tissue-specific effects must also be considered (Bianchi et al., 2020; Madia et al., 2021; Arner and Rathmell, 2022; Doe et al., 2022).

In 2020, the European Commission adopted its Chemicals Strategy for Sustainability (EC, 2020) with the main objective of promoting innovation for safe and sustainable chemicals, and improving the protection of human health and the environment from hazardous chemicals. This led to the establishment of the EU-funded Partnership for the Assessment of Risk from Chemicals (PARC) (Marx-Stoelting et al., 2023). This partnership consists of several sub-projects and sub-tasks. In the current sub-task project, we aim to provide appropriate assays for efficient and improved hazard assessment of NGTxCs, in order to support a mechanism-based next-generation risk assessment (NGRA) of chemical carcinogens.

To achieve this goal, a consortium of 13 partners across Europe has been established within the PARC project. Each partner contributes with its own expertise and knowledge in assay development that are compiled into a comprehensive strategy to identify and predict the carcinogenic potential of NGTxCs. The selected assays address a range of endpoints relevant to human carcinogenesis, and cover a broad spectrum of MoA that fit into the concept of hallmarks of cancer.

**The objective:** *improve and develop NAMs for the identification and the characterization of NGTxCs and the establishment of a reliable and efficient testing strategy for development of a safety assessment toolbox for NGRA.*

In light of the limitations of the 2-year rodent bioassay, which under some regulations is the required test for the identification and characterization of carcinogens, there is an urgent need for an efficient and reliable *in silico* and *in vitro* testing battery. Several past and ongoing European research initiatives, not exclusively dedicated to NGTxCs detection, aim to develop new *in vitro* toxicity test methods. These include the EU-ToxRisk and RISK-HUNT3R projects as well as the ASPIS (https://aspis-cluster.eu/) and EURION (https://eurion-cluster.eu/) project clusters. Over the last years, a number of new and improved methods have become available that might play a pivotal role in the development of a regulatory acceptable integrated approaches for testing and assessment (IATA) for NGTxCs. In 2020, an OECD expert group developed an IATA for NGTxCs and published a consensus paper describing the overarching IATA, with the molecular initiating events (MIE) of cellular metabolism and receptor interactions, followed by the early key events (KEs) of inflammation and immune dysfunction, mitotic signaling, and cell injury, leading to (sustained) proliferation, morphological transformation and tumor formation (Jacobs et al., 2020). Several assay evaluations and reviews have been conducted and published in relation to epigenetics (Desaulniers et al., 2021), cell transformation (Colacci et al., 2023), gap junction (Sovadinova et al., 2021), gene signaling (Oku et al., 2022), cell proliferation and oxidative stress (Veltman et al., 2023). These publications provide details on the next steps needed to facilitate developments that will have regulatory relevance for the safety assessment of NGTxCs.

Our project aims to provide a comparison of the different selected methods to check how they could be applied and how they may complement in the context of an IATA. In this context, the project can also help to improve knowledge on the relationships between specific toxicity mechanisms and adverse outcome pathways (AOPs) leading to cancer. AOPs correspond to chains of events starting from a MIE, then describing all the steps involving KEs at different biological levels (cellular, tissue, organism) leading to an adverse outcome (toxic impact or pathology). For the *in vitro* identification of NGTxCs, it is mandatory that the developed methods cover MIEs and/or KEs associated with hallmarks of cancer. In this context, AOPs can be a useful framework for mapping aspects of these complex relationships of carcinogenesis.

In this project, the partners provide a wide variety of methods using model systems of increasing complexity, from 2D human cell lines to advanced 3D human systems and zebrafish embryos models. These innovative human-relevant NAMs focus on organs (liver, breast and colon) commonly affected by chemical carcinogens, and include state-of-the-art transcriptomic approaches, cell painting, high-content image analysis and high-throughput-compatible reporter systems. The assays address well established and relevant mechanisms involved in carcinogenesis, (Table 1). The challenge here is to sufficiently capture the high complexity of NGTxCs triggered carcinogenesis with a manageable testing battery covering key features of human cancer (Table 1) (Hanahan, 2022). Moreover, factors supporting tumor initiation and progression as oxidative stress, activation of nuclear receptors and (tissue-specific) cytotoxicity leading to continuous regenerative proliferation are important mechanisms, but do not count to the hallmarks of cancer themselves. Excessive oxidative stress is known to play an important role in tumorigenesis by causing oxidative DNA damage that can directly lead to mutations. On the other hand, oxidative stress can also stimulate proliferation and epithelial-mesenchymal transition (EMT), leading to cancer progression through metastatic processes, and can be associated with inflammatory responses and cytotoxicity (Heusinkveld et al., 2020a; Hayes et al., 2020).

**TABLE 1:** Overview of the different cell types, complexity levels, exposure scenarios and endpoints addressed by the various partners of the NGTxC project. A = acute, R = repeat.

Partner	Cell/Tissue models	2D/3D	Treatment	Endpoints								
				Genomic instability	Proliferation	Cytotoxicity	Oxidative stress	NR-activation	Migration	Phenotypic changes	Inflammation	Epigenetic changes
ANSES	Liver	2D/3D	A/R		•	•	•	•		•	•	•
BfR	Breast, Liver, Colon	2D	A/R		•	•		•	•	•		
INRAE	Multicellular liver	3D	A/R	•	•	•	•			•	•	
INSERM	Breast, Adipocytes	2D/co-culture	A		•	•			•	•		
IRFMN	NA	NA	NA	•				•				
NIB	Liver	3D	R	•	•	•	•	•		•		•
NILU	Breast	2D/3D	A		•	•	•				•	
RECETOX	Liver	2D	A		•	•						
RIVM	Zebrafish	NA	A/R		•		•					
RPTU	Colon	2D	A/R	•	•	•	•		•			
UL-LACDR	Breast, Liver	2D	A		•	•	•	•	•	•	•	
UNAV	Liver	2D/3D	A/R	•		•	•					

ANSES: french agency for food, Environmental and Occupational Health and Safety, BfR: german federal institute for risk assessment, INRAE: national research institute for agriculture, Food and the Environment, France INSERM: national institute for health and medical research, France, IRFMN: istituto di ricerche farmacologiche mario negri, Italy, NIB: national institute of biology, Slovenia, NILU, the climate and environmental research institute, Health Effects Laboratory, RECETOX: research centre for toxic compounds in the environment czech republic, RIVM: national institute for public health and the environment, the Netherlands, RPTU: Rheinland-Pfälzische Technische Universität, Germany, UL-LACDR: leiden academic centre for drug research, the Netherlands, UNAV: University of Navarra, Spain.

Interestingly, methods based on Nrf2 gene reporter cell lines, a key mediator of oxidative stress response, as well as a direct assay for measuring reactive oxygen species (ROS) content have already been accepted by the OECD as test guideline (TG) TG 442D and TG 495, with a focus on skin sensitization and phototoxicity, respectively. Estrogen receptor activation is a common therapeutic target, closely linked to the proliferation of endometrial, colorectal, prostate and breast cancer, but also associated with metastasis, EMT and epigenetic changes (Garcia-Martinez et al., 2021). Similarly, the role of AhR-mediated functions in cancer development is not limited to the generation of carcinogenic metabolites, but also feeds into various other cancer-related processes, including cell migration, cell transformation, inflammation, and epigenetic processes (Larigot et al., 2018; Larigot et al., 2022). Finally, sustained cytotoxicity has been shown to be an important mechanism of action for carcinogenic chemicals, particularly inducing regenerative proliferation (Hernandez et al., 2009). Thus, the endpoints addressed by the consortium partners are related to several hallmarks of cancer. As the same endpoint is addressed by different methods, their respective applicability will be established by comparing the responses of appropriate reference substances. Moreover, the comparison will also investigate the relevance of cell models and type of exposure methods in effects of carcinogenic chemicals. The variety of models and methods in this project should facilitate the development of an efficient and reliable test battery to identify NGTxCs and analyze their responses. Experimental approaches will be complemented by the optimization of *in silico* tools for the identification of NGTxCs.

A large set of reference compounds (positive or negative for carcinogenicity), including some prioritized PARC compounds (toxins and bisphenol analogues), will be tested, allowing chemical specific comparison of the different methods. The number and chemical classes of reference chemicals to be tested for the various mode of actions will depend upon the availability of reliable human-relevant data, including rodent bioassay data. The sensitivity and specificity of the various systems as well as their complementarity in detecting the different mechanisms involved in non-genotoxic carcinogenicity will be supplied.

Next, we will describe in more detail the methods applied in the project by the individual consortium partners.

### ANSES: High content analysis of oxidative stress, nuclear receptor translocation, epigenetic markers, inflammatory responses, and mitochondrial metabolism in liver cell models

High-content analysis (HCA) is the application of automated high-resolution microscopy combined with quantitative image analysis for cell-based or organoid-based assays. This multiparametric image-based approach is capable of quantifying a large number of features at single cell and population levels. In addition, the high throughput nature of the approach enables a large number of chemicals or endpoints to be tested in a single experiment. Furthermore, HCA can be used for the analysis of live and fixed cells, which makes this approach an important *in vitro* method for toxicological and mechanistic studies needed to predict NGTxCs (Li and Xia, 2019). Through the use of antibodies or fluorescent probes specific for cellular processes, this system is well suited for the detection of different hallmarks of cancer, including oxidative stress, nuclear receptor translocation, epigenetic markers, inflammatory responses, and mitochondrial metabolism. HCA therefore represents a promising *in vitro* approach to detect NGTxCs. In this context, a HCA approach to assess potential mode of action of NGTxC compounds using the human bi-potent progenitor HepaRG cell line in 2D and more complex 3D spheroid models will be applied (Marion et al., 2010).

### BfR: Identification of NGTxCs by phenotypic screening for carcinogenic effects on cell adhesion, migration, proliferation, and mitochondrial dysfunction

The BfR will apply phenotypic screening methods in order to investigate and group compounds according to their modes of action. The first model, called E-morph assay, is based on ER-alpha expressing MCF7 breast cancer cells and evaluates the changes in adhesion junction (AJ) organization as an indicator for anti-estrogenic or estrogenic effects (Kornhuber et al., 2021). The Estrogen-dependent changes in the AJ directly influence cell stiffness and motility, two functions that are highly relevant for cancer progression, in particular in respect to progression from benign to malignant tumors or metastasis and address the relevant endpoints of phenotypic changes-migration and nuclear receptor activation at the same time By applying a cytoplasmic stain on the parental cells or by using an E-Cadherin-EGFP-expressing reporter cell line (de Beco et al., 2009; Klutzny et al., 2022), the appearance of the plasma membrane can be quantified by high content imaging. Depending on the assay set-up specific competitive compounds can be included allowing to screen for (anti-) estrogenic or (anti-) glucocorticoid compounds.

In our second model, cell-painting techniques (Bray et al., 2016) will be applied in order to generate compound-specific morphological profiles. In this assay, the breast cancer cell line MCF7 and the non-tumor cell line hTERT-HME1 are labeled with 6 different stains, followed by high content imaging and computational image analysis. The extraction of up to 50 features (e.g., size, shape, intensity, texture) allows the generation of compound-specific data. The direct comparison of a rather normal, immortalized cell line with a cancer cell line might also facilitate the detection of specific effects on the process of tumor progression, also in respect to proliferation, oxidative stress and cytotoxicity. Based on the assumption that compounds with similar profiles share common modes of action (Fetz et al., 2016), the applicability to identify carcinogenic compounds will be evaluated. Within this project, it will also be tested if the classical cell painting set up or another dye-set allowing the detection of different cellular structures can contribute best in the discrimination of such compounds. In parallel, the effects on the composition of the extracellular matrix (ECM) will be analyzed. Changes in the composition of the extracellular matrix have long been known to play an important role in the activation of “silent” neoplasia (Bissell and Hines, 2011) and, thus, a thorough analysis of chemically induced changes in ECM composition will also provide a better mechanistic insight into the effects of potential carcinogens on cellular migration and metastasis.

### INRAE: Distinguishing genotoxic carcinogens from NGTxCs

In the EU, carcinogenicity studies are required for new active substances in the pesticides (1107/2009) and biocide (528/2012) regulations, whereas they are only required for high tonnage industrial chemicals under REACH (1907/2006). They can be waived if no evidence for hyperplasia or pre-neoplastic lesions are detected in repeated-dose studies and the substance is found non-mutagenic in genetic toxicity tests. Cancer has long been known to be induced by mutations, a molecular process that led to the development of the classical battery of genotoxicity tests available to detect chemicals that induce gene mutations (OECD TG 471, 476, 490) and chromosomal aberrations (OECD TG 473, 484). Although the sensitivity of this battery of the current regular genotoxicity assays is high, the specificity is rather low leading to a high false-positive rate compared with *in vivo* data, especially with the mammalian cell-based assays.

The methodology proposed by the INRAE team allows an evaluation of genotoxicity using two biomarkers, phosphorylated histones H2AX (γH2AX) and H3 (pH3), specific for clastogenic and aneugenic chemicals, respectively. The γH2AX biomarker is also related to genomic instability, is detected in cancerous cells and clinically used in cancer biology (Palla et al., 2017). The γH2AX/pH3 method was developed and pre-validated on 2D cell models and demonstrated a predictivity higher than 90% (Kopp et al., 2019). Moreover, some NGTxCs chemicals were detected with this method, indicating that this assay may permit to detect genotoxicity of some NGTxCs not revealed previously through the regular OECD genotoxicity assays (Kopp et al., 2019). In the PARC project, the assay will be further developed with a 3D multicellular liver model. The spheroids will include different cell types: the HepaRG hepatoma cell line, Kupffer cells and hepatic stellate cells (Yan et al., 2021). Repeated chemical treatment over numerous days will be used to take into account the real human exposure more efficiently. Additional specific biomarkers of cytotoxicity, oxidative stress, proliferation and inflammation will be included to discern some specific chemical toxic MoA. In the same idea, cell painting experiments will be performed on this 3D model to detect phenotypic changes induced by the compounds.

### INSERM: Analysis of cell migration

The methodology proposed by the METATOX team (Inserm T3S) allows the real-time monitoring of proliferative, morphologicaly and migratory capacities of breast cancer cells in a context that mimics the tumor microenvironment. Additionally, a change in the cellular morphology provides a qualitative indication of potential cytotoxicity (to be then confirmed using other specific tests). The system is based on the co-culture of MCF7 human breast cancer cells and hMADS human pre-adipocytes in multi-well plates equipped with a permeable membrane insert that allows communication between the two cell types. We already demonstrated that by exposing this model to Seveso dioxin or a mixture extracted from cigarette smoke, the phenotype of the cancer cells was modified, with the setting up of a process suggesting an epithelial-mesenchymal transition (EMT), the first step in the formation of metastasis (Koual et al., 2021; Benoit et al., 2023). Changes in cell phenotype (morphology and migration) can be followed in real-time using the xCELLigence system. In case of modification upon exposure to a chemical, morphological changes can be further characterized using microscopy and expression profile analysis for biomarkers of tumor progression taking proliferation and cytotoxicity into account. This model has the great advantage of mimicking the early stages of metastatic cell formation and appears relevant to study the influence of NGTxCs that may promote metastasis, which is involved in up to 90% of cancer mortality.

### IRFMN: In silico identification of NGTxCs

At the basis of the *in silico* models there is the possibility of anticipating the activity of a compound starting from its chemical structure. It is known that, in fact, certain physico-chemical properties, such as the melting point, are related to the chemical structure. Similarly, certain types of toxicities are predicted with QSAR (Quantitative Structure-Activity Relationship) models, too. As initial steps, we collect information on the feasible *in silico* models related to carcinogenicity (Benigni et al., 2008; Fjodorova et al., 2010; Benfenati and Gini, 2013; Benigni et al., 2013; Golbamaki et al., 2016; Toma et al., 2020) and associated endpoints, such as genotoxicity (Roncaglioni et al., 2008; Baderna et al., 2020; Van Bossuyt et al., 2020) and nuclear receptor activity. Indeed, there are many QSAR models freely available, for instance within the platform of *in silico* tools, VEGAHUB (https://www.vegahub.eu/). These tools can be used on the selected compounds, to evaluate the carcinogenicity potential and the binding to specific nuclear receptors (https://www.vegahub.eu/portfolio-item/vega-nrmea/), for instance. The analysis of the predictivity of the *in silico* approach towards this kind of biological activity will be examined in depth developing new *ad hoc* models starting from big sets of data (e.g., ICE–Integrated Chemical Environmental of NTP) related to the key characteristics of carcinogens. An additional activity is using the tools for the so called read-across approach (https://www.vegahub.eu/portfolio-item/vera/) (Vigano et al., 2022). In this case, the model explores the similarity between the target substance and related compounds with experimental data.

### NIB: High Content analysis of genotoxicity, epigenetic changes, AhR activation, stress response and cell proliferation in liver spheroids

A multi-labeling approach using specific antibodies and stains and the use of flow cytometry to simultaneously detect multiple endpoints in the same cell and cell population represents a HCA tool. The effects of chemicals, including NGTxCs, on cell proliferation will be studied by detecting fluorochrome signals for proliferation markers (e.g., Ki67, PCNA) and by analyzing the distribution of cells in the cell cycle. In addition, other hallmarks of carcinogenesis will be investigated simultaneously, including genomic instability by detecting DNA damage (γH2AX marker) reflecting clastogenic activity, and mitotic cells (histone H3-positive cells) reflecting aneugenic activity, oxidative stress, nuclear receptor activation (AhR), and epigenetic modifications of histones (e.g., H3K27me3, H3K9ac, and H3K9me2) that serve as predictors of carcinogenicity (Stampar et al., 2022). The effects on cell growth and proliferation will be assessed using the MTS assay.

Toxicogenomics, the application of gene expression analysis techniques in toxicological studies, identifies global changes in gene expression associated with a toxicological outcome and contributes to the refinement of toxicity and carcinogenicity testing. It provides supporting mechanistically based data on the molecular pathways underlying the apical effects (Ellinger-Ziegelbauer et al., 2005). Recently, gene expression profiling has been proposed as a useful tool for hazard identification and human health risk assessment, providing not only qualitative, but also quantitative information related to relevant mechanisms of action induced by the stressors (Corvi and Madia, 2017). It is well known that stressors with similar biological activities (e.g., oxidative stress inducers, genotoxic carcinogens/NGTxCs) can deregulate the expression of specific genes, which can therefore be used to distinguish different molecular mechanisms of action of the stressors under study (biomarkers of exposure or effect). Using a combination of multi-labeling and toxicogenomics approaches, the mechanisms of action of NGTxCs will be investigated in a hepatic *in vitro* 3D cell model (spheroids) developed from human hepatocellular carcinoma (HepG2) cells. The advantage of spheroids over traditional monolayer cultures is that they more closely resemble tissue cell organization and therefore better mimic the *in vivo* microenvironment and provide more predictive data for human exposure (Stampar et al., 2020).

### NILU: *In vitro* cytotoxicity, oxidative stress (ROS), proliferation and inflammatory markers

Breast cancer (BC) is one of the most common cancer type in the world and the second leading cause of cancer deaths among women (Bray et al., 2018). Exposure to environmental chemicals and pollutants have been linked to BC incidence (Kortenkamp, 2006; Bonefeld-Jorgensen et al., 2011). BC mortality is associated mainly with the development of metastases (Ruscitto et al., 2022). Therefore, identification of the mechanisms involved in metastasis formation is of high importance. The signaling pathways involved in metastatic tumor cells emergence and progression are increasingly linked to exposure to environmental chemicals and pollutants (Koual et al., 2020; Gabet et al., 2021; Kay et al., 2022). Oxidative stress is one of the best documented mechanisms for carcinogenesis, together with impacts on cell cycle progression (Martínez-Reyes and Chandel, 2021; Dharshini et al., 2023). Environmental pollutants may induce cell damage by induction of ROS and oxidative stress, which can trigger an inflammatory response which may also evolve into a chronic inflammation (Dharshini et al., 2023). Inflammation has been linked to various steps in tumorigenesis, including cellular transformation, promotion, survival, proliferation, invasion, angiogenesis, and metastasis. NILU´s contribution to this project will focus on breast cancer and breast cancer cell models. We will study oxidative stress and ROS production as an early KE in carcinogenesis, as well as induction of relevant pro-inflammatory cytokines (ELISA) (Esquivel-Velazquez et al., 2015). Further, we will study cell proliferation. One of the promising advanced *in vitro* cellular models which mimic more closely the *in vivo* situation, is the 3D spheroids models. Different cell lines with features representing different BC subtypes, JIMT-1, MDA-MB-231 and T-47D have been used to establish advanced 3D multicellular aggregates. NILU will focus on one of these cell lines and apply the above mentioned endpoints. Both 2D and 3D models of JIMT-1 cells will be used, and their sensitivity compared. In addition to the use of cancer cell lines, the use of normal cell lines should also be considered, e.g., the epithelial MCF-10A cell line. These cells are subline of spontaneously immortalized human breast epithelial cells derived from human fibrocystic mammary tissue with characteristics of normal breast epithelium, which make the MCF-10A a valuable *in vitro* model to study normal breast cell function and determine the potential of environmental chemicals, including NGTxCs, to induce tumor transformation.

### RIVM: Analysis of oxidative stress responses in zebrafish

RIVM’s contribution to this project is focused on oxidative stress. Oxidative stress, resulting from an imbalance between the generation of oxidants and their scavenging by antioxidants, may lead to sustained cytotoxicity and regenerative proliferation and is therefore a MoA relevant for carcinogenesis (Hanahan and Weinberg, 2000; Hanahan and Weinberg, 2011; Leuthold et al., 2019; Hanahan, 2022). Oxidative stress can be induced through endogenous processes, but also upon exposure to chemical substances (Heusinkveld et al., 2020b; Veltman et al., 2023). This is known to promote carcinogenesis through DNA damage and impaired repair, as well as through indirect actions influencing homeostasis and signaling (Klaunig, 2018). Additionally, ROS play a role in numerous stages of the multistep carcinogenic process (Weinberg et al., 2019).

RIVM will use zebrafish embryos as whole-organism model for measuring oxidative stress for a diverse set of substances. The added value of this model for toxicity testing of both single substances and chemical mixtures, as well as MoA identification has been shown by us and others (Leuthold et al., 2019; Heusinkveld et al., 2020b; Bauer et al., 2021; Van Der Ven et al., 2022). Various parameters for oxidative stress will be measured, including ROS levels, lipid peroxidation and changes in gene expression. For this purpose, both fluorophore-based techniques in wild-type zebrafish and transgenic reporter lines are available, responsive to activation of the Nrf2 pathway allowing for microscopic determination of oxidative stress on a multi-organ level in live embryos. The rationale for using zebrafish embryos is to generate data in a more complex test system, complementary to other models available to the project. In particular, the zebrafish can provide data for higher-tier key events in AOP approaches (organ-organism levels) that are not covered by (complex) cellular models.

### RECETOX: Analysis of intercellular communication

The RECETOX team will explore the applicability of the scrape loading-dye transfer (SL-DT) assay (Dydowiczova et al., 2020) assessing gap junction intercellular communication (GJIC) *in vitro* using rodent and human liver cells to distinguish between known NGTxCs and non-carcinogens. GJIC is a vital process in multicellular animals, facilitating the exchange of ions and molecules between cells and coordinating tissue actions (Nielsen et al., 2012; Zefferino et al., 2019). Disruption of GJIC has been a well-recognized but overlooked hallmark of NGTxCs for a long time (Trosko et al., 2004; Aasen et al., 2016; Jacobs et al., 2016; Zefferino et al., 2019; Jacobs et al., 2020). In the recently proposed IATA for identification of NGTxCs, GJIC has been linked to pivotal key events of mitogenic signaling and cell injury (Jacobs et al., 2020), thus related to the endpoints of cell proliferation and cytotoxicity. However, there are no validated methods for GJIC assessment. While several methods can be used to evaluate the dysregulation of GJIC, many have limited throughput and require specialized equipment (Sovadinova et al., 2021). The SL-DT assay is the most commonly used *in vitro* method for evaluating the impact of toxicants or carcinogens on GJIC with a proven potential for high-content analysis and screening (HCA/HCS) (Dydowiczova et al., 2020; Sovadinova et al., 2021). The SL-DT assay has been widely employed to study alterations in GJIC caused by hundreds of chemicals, including NGTxC. Overall, the sensitivity of the SL-DT assay to predict IARC carcinogens (Group 1, 2A, or 2B) is 77% (Sovadinova et al., 2021). However, most of the published data were generated using a rat liver epithelial cell line (WB-F344), and negative compounds (non-carcinogens) have not been widely assessed. These issues will be addressed in this project by testing a common set of reference compounds using the multiparametric SL-DT assay allowing assessment of GJIC along with cell proliferation and cytotoxicity (Dydowiczova et al., 2020). The results obtained in WB-F344 cells will be validated the results using human cells. This will help evaluate the potential of the multiparametric SL-DT assay as a screening/testing tool for NGTxCs within integrated testing approaches.

### RPTU: Identification of NGTxCs using human colon cell models

Non-malignant human colonic epithelial cells (HCEC) (Roig et al., 2010) and HCT116 human colorectal cancer (CRC) cells (Ahmed et al., 2013) have already being used to study dietary chemical carcinogens, bacterial toxins as well as drug candidates in the context of CRC (Mimmler et al., 2016; Seiwert et al., 2017; Dorsam et al., 2018; Seiwert et al., 2020; Arnold et al., 2022). To identify NGTxCs, a test battery covering the following endpoints will be applied: cell proliferation, formation of ROS, EMT, cell migration and genotoxicity. The latter endpoint is based upon the sensitive detection of γH2AX as established DNA damage marker (Kinner et al., 2008) using InCell Western or immunofluorescence methodology, which will help to distinguish genotoxic from non-genotoxic compounds. Increased cell proliferation is a well-described hallmark of cancer (Hanahan, 2022) and known to be triggered by various NGTxCs. Effects on cell growth and proliferation will be first assessed using the Alamar Blue cell viability assay (Vieira-da-Silva and Castanho, 2023), allowing for extensive concentration-range finding studies in a high-throughput manner. This will be complemented by the 5-ethynyl-2′-deoxyuridine (EdU) assay, which measures the incorporation of EdU into nascent DNA during replication (Salic and Mitchison, 2008) and thus correlates with cell proliferation. EdU incorporation is visualized via Click chemistry using fluorescent azides, such as 6-fluorescein azide, followed by fluorescence microscopy or flow cytometry in low-to-medium throughput.

The induction of oxidative stress will be analyzed as a further endpoint using the cell-permeable probe CM-H_2_DCFDA, which is converted intracellularly by ROS into a fluorescent dye (Kalyanaraman et al., 2012). This can be measured in a multi-well plate reader using an additional Hoechst staining for normalization to cell counts. Depending on its level, spatiotemporal formation and chemical nature, oxidative stress contributes to carcinogenesis via both non-genotoxic and genotoxic mechanisms (Sies and Jones, 2020). Another important endpoint in the context of metastasis is EMT. This process involves the activation of specific transcription factors, leading to the reorganization of the actin cytoskeleton and degradation of extracellular matrix as a prerequisite for migration and invasion (Kalluri and Weinberg, 2009). Here, the F-actin will be stained using a fluorescent phalloidin probe, which will be combined with the detection of E-cadherin as a marker of epithelial morphology that decreases upon EMT. Both markers are then visualized by fluorescence microscopy, but may alternatively be measured using a multi-well plate reader to adjust to more samples.

Finally, cell migration as pivotal step during the metastasis of cancer cells will be studied using the so-called wound healing assay that is also termed scratch assay (Vang Mouritzen and Jenssen, 2018). To this end, confluent cells with a scratch/gap are exposed to the compound of interest and cell migration is then monitored as gap closure via microscopy (endpoint measurement), thus somewhat limiting the number of samples to be analyzed.

### UKHSA: Augmentation of the CYP induction test method for use in the NGTxC IATA and validation management for the cell transformation Assay using transcriptomics: the transformics assay

UKHSA will provide input on the further development of the HepaRG CYP induction test method (Bernasconi et al., 2019) (Jacobs et al., 2022), to support the NGTxC IATA (Jacobs et al., 2020) and will be implicated for the (pre)validation of promising test methods that address the outstanding needs as identified in the work of the OECD NGTxC IATA expert group, specifically here in relation to cell transformation (Colacci et al., 2023) and gap junction (Sovadinova et al., 2021) in the first instance. A regulatory framework for the IATA for NGTxC developed with ECHA will also be provided (Louekari et al. paper in prep), this will assist in supporting the regulatory targeted assay development.

### UL-LACDR: High-Throughput screening for oxidative stress as well as mitogenic and inflammatory signaling

UL-LACDR focuses on quantitative, mechanism-based, and human-relevant hazard characterization, employing an AOP/AON conceptual thinking in direct relation to the hallmarks of cancer concepts and taking advantage of high-throughput modular assays using automated confocal microscopy and high throughput transcriptomics. This allows for testing redundancy and validation of methodologies whilst permitting constant improvement and innovation of integrated testing strategies. The approach will follow a tiered testing strategy that includes high-content 2D and 3D cell-based imaging platforms for next-generation risk assessment of NGTxCs. In the context of this project, the focus will be on mammary gland and liver, two of the most critical target organs for chemically induced carcinogenesis in 2-year rodent cancer bioassays (Sistare et al., 2011). Pivotal key events in the proposed integrated approach to testing and assessment (IATA) of NGTxCs (Jacobs et al., 2020) will be addressed, in particular oxidative stress, inflammatory signaling and mitogenic signaling. Since several known human NGTxCs target estrogen receptor signaling, fluorescent BAC reporters for ERα-induced proliferation and cell cycle progression in human ERα-positive MCF7 breast cancer cells will be included (Duijndam et al., 2021; Duijndam et al., 2022), as well as a panel of CRISPR-based endogenously tagged fluorescent human induced pluripotent stem cell reporter lines that can be differentiated to relevant lineages including progenitor cells of mammary epithelial cells and hepatocytes. These reporters cover diverse critical cellular signaling responses that are essential in cancer development. Using validation sets of chemical compounds with known toxic effects, the reporter lines will be evaluated with high content live cell confocal imaging for sensitivity and specificity in the detection of specific types of stress-response in different target organ lineages, in 2D or 3D. This includes the analysis of general markers of cytotoxicity and effects on proliferation, migration and other cellular phenotypes. The imaging-based approaches will be complemented by high throughput targeted mRNA sequencing as well as high throughput single-cell sequencing using TempO-Seq (https://www.biospyder.com) or BART-seq (Uzbas et al., 2019), to get broader insight in AOP activation and thresholds of carcinogenic events.

### UNAV: Detection of repairable DNA lesions induced by NGTxCs

Genotoxic carcinogens are compounds that give a positive response in classical batteries of genotoxicity testing, which includes *in vitro* and *in vivo* assays that mainly detect point mutations or chromosomal aberrations [e.g., ICH (ICH, 2008) and EFSA (EFSA, 2011)], and in the *in vivo* 2-years carcinogenicity studies. Compounds that induce DNA lesions that are completely repaired may not be detected by the genotoxicity battery.

On the other hand, NGTxCs are compounds that induce tumors in the *in vivo* carcinogenicity studies but are negative in the aforementioned genotoxicity testing batteries. However, the current regulatory genotoxicity testing strategies are mainly focused on point mutations or chromosomal aberrations. A key question is whether the presence of repairable DNA lesions could be a marker for some NGTxCs. To explore this option the detection of DNA lesions, also called pre-mutagenic lesions, that cause genomic instability, will be carried out *in vitro* using different versions of the alkaline comet assay.

The standard version of the alkaline comet assay detects DNA strand breaks and alkali labile sites (ALS), such as abasic sites, at a single cell level (Collins et al., 2023). The assay is used in genotoxicity testing, in both *in vitro* and *in vivo* models. The *in vivo* version is included in the ICH and EFSA genotoxicity testing strategies (ICH, 2008; EFSA, 2011); its OECD test guideline was published in 2014 and reviewed in 2016 (OECD, 2016c). Indeed, this is the only assay included in the standard testing strategies that does not detect point mutations or chromosomal aberrations, but DNA strand breaks and ALS.

On the other hand, the comet assay has been modified to detect specific DNA lesions (Collins et al., 2023) such as altered bases and it is widely used for the detection of oxidized bases, a marker of oxidative stress. Oxidized DNA bases can be induced directly by a compound or indirectly through a physiological toxic response. In PARC the alkaline comet assay will be used in its standard version but also combined with the enzyme Fpg for the detection of oxidized purines and with the enzyme hAAG for the detection of alkylated DNA bases (Muruzabal et al., 2020; Muruzabal et al., 2021). These three versions of the assay will be applied in 2D and 3D HepG2 human liver cells after acute and repeated exposure. Cytotoxicity will be also measured in the same exposure conditions.

## Concluding remarks

In the course of this project, a wide variety of already established methods will be optimized to test the effects of NGTxCs on different KEs known to be involved in carcinogenesis. In addition, new methods and NAMs will be developed and tested to complement existing methods with respect to the various modes of action of NGTxCs. Other work packages from PARC focus on the development of IATAs and AOPs for genotoxic and NGTxCs. It will be important to align this work with the efforts of the OECD that has established an expert group to evaluate the currently available NAMs suitable for the published NGTxC IATA (Jacobs et al., 2020). Most importantly, several PARC projects are addressing toxicological endpoints that are directly related to (non-)genotoxic carcinogenesis, in particular immunotoxicity, inflammation and metabolic endocrine disruption. In the future, NAMs developed in these specific PARC projects as well as new methods currently being developed in EU Horizon 2020 projects, in particular in the EURION and ASPIS clusters, but also methods that are already established and are undergoing validation, will be taken into account. It is important to ensure that there is sufficient overlap in the selection of compounds tested to allow, at a later stage, a comprehensive analysis of various combinations of these methods in testing strategies. In combination with the assays developed in additional PARC and Horizon 2020 projects, test methods for several of the defined hallmarks of cancer will be (further) developed. Comprehensive coverage of all the hallmarks of cancer will not be possible in the PARC project alone or even in cooperation with ongoing EU projects, including processes such as de- or trans-differentiation or the influence of the microbiome.

This project provides a unique opportunity to directly compare a wide variety of methods and approaches, and the results will feed into other projects and work packages to support the development of AOPs and IATAs, in close cooperation with parallel activities in Europe and the OECD. This work will facilitate the development of more efficient and human-relevant assessment of chemicals for potential non-genotoxic carcinogenic activities by developing NAMs for identification of NGTxCs, mapping KEs for further developing AOPs and IATA for carcinogenicity, and contributing to the safety assessment toolbox to support NGRA of carcinogens (Bischoff et al., 2020).
